# Exploratory Study on the Prevalence of Peripheral Artery Disease in Asymptomatic Patients With Diabetes and Hypertension in a Family Health Unit

**DOI:** 10.7759/cureus.87185

**Published:** 2025-07-02

**Authors:** Mariana C Castro, Pedro Gomes, Carolina Roldão, Bruno Rei, Ana Cecília Chaves, Ana Rita Laranjeiro, Isabel Fragoso, Marta Cardoso, Inês Rosendo

**Affiliations:** 1 Family Medicine, USF Araceti, Coimbra, PRT; 2 Public Health Unit, Unidade Local de Saúde Gaia e Espinho, Vila Nova de Gaia, PRT; 3 Faculdade De Medicina De Coimbra, Universidade de Coimbra, Coimbra, PRT

**Keywords:** ankle-brachial index, cardiovascular risk, diabetes mellitus, lower extremity artery disease, systemic arterial hypertension

## Abstract

Introduction: Lower extremity artery disease (LEAD) represents a manifestation of atherosclerosis, with the majority of affected individuals remaining asymptomatic. Whether asymptomatic or not, LEAD is associated with a three- to six-fold increased risk of cardiovascular mortality, underscoring the importance of early intervention. The ankle-brachial index (ABI) is a recommended screening tool for LEAD, with a diagnostic threshold defined as an ABI of ≤0.90. Patients with arterial hypertension and/or diabetes mellitus (DM) are at elevated risk for developing LEAD, yet systematic screening for this condition is lacking in this population. The primary objective was to determine the prevalence of asymptomatic LEAD in patients with arterial hypertension and/or DM, while the secondary objective was to identify the main predictors of LEAD in this population.

Materials and methods: This was an exploratory, observational, cross-sectional study involving a random sample of patients with DM and/or arterial hypertension enrolled in a family health unit (FHU), aged 50 to 66 years, without very high cardiovascular risk (CVR) or symptoms of LEAD. ABI was measured, and LEAD was diagnosed if ABI ≤0.90. Statistical analyses were performed using SPSS® software.

Results: The study included 146 patients, of whom 134 had arterial hypertension, and 68 had DM, with a mean age of 59.5±4.2 years. Eight patients were diagnosed with LEAD, yielding a prevalence of 5.5%. Statistically significant differences were observed between the groups with and without LEAD in the following variables: abdominal circumference (p=0.043), glomerular filtration rate (GFR) using Cockcroft-Gault (CG) equation (p=0.024), duration of arterial hypertension (p=0.024), and LDL levels (p=0.014), with higher medians in the LEAD group.

Discussion: There are several studies calculating the prevalence of LEAD, but variations in study populations make direct comparisons challenging. The LEAD group showed higher abdominal circumference, longer hypertension duration, and elevated LDL cholesterol, consistent with existing literature. However, the significantly higher GFR observed in this group using the CG equation was unexpected. This may be explained by the inclusion of body weight in the CG formula, which can overestimate GFR in individuals with central obesity due to increased fat mass rather than increased renal function. Conversely, the Chronic Kidney Disease Epidemiology Collaboration (CKD-EPI) equation, which does not incorporate body weight, showed no significant difference in GFR between groups, supporting the hypothesis that CG estimates were biased by body composition. Regarding limitations, this study possibly had information and inter-observer bias, a lack of confirmation of diagnosis by imaging study, as well as an under-representation of smokers, an important risk factor for LEAD, which may have influenced the calculation of prevalence.

Conclusion: The prevalence of asymptomatic LEAD in patients with DM and/or arterial hypertension within our sample was 5.5%. The results suggest that patients with higher abdominal circumference, longer duration of arterial hypertension, and elevated LDL are at increased risk for LEAD, supporting the prioritization of this population for screening. However, further studies are essential to better define the optimal target population for LEAD screening in Portugal.

## Introduction

Lower extremity arterial disease (LEAD) is most commonly caused by atherosclerosis [1] and results from the partial or complete obstruction of one or more arteries in the lower limbs [2].

Suffering from LEAD is also an indicator of systemic atherosclerosis in other vascular territories, such as the coronary, carotid, and cerebrovascular arteries, which may eventually lead to major events such as acute myocardial infarction or stroke [2]. Regardless of the presence of symptoms, it is associated with a three to six times higher risk of death from cardiovascular causes [3], which is why patients benefit from the implementation of cardiovascular risk (CVR) prevention and disease progression control strategies, primarily through the rigorous management of CVR factors [4].

In most cases, LEAD is asymptomatic, which may be related to the physical limitations described by patients due to their comorbidities (such as heart failure) and/or reduced sensitivity, as seen, for example, in diabetic neuropathy [5]. This leads to underdiagnosis, delayed diagnosis, and consequently, undertreatment and/or late treatment of the disease [5].

The ankle-brachial index (ABI) is a simple, non-invasive method for screening and diagnosing LEAD [4], with a sensitivity of 80% and specificity of 95% [6,7]. An ABI of 0.90 or lower indicates LEAD, a condition linked to a significantly elevated CVR. This should prompt the initiation of secondary prevention measures [1,8].

LEAD has been associated with several classic CVR factors, particularly advanced age, smoking, hypertension, dyslipidemia, and type 2 diabetes mellitus (DM) [2].

Hypertension is likely the most common risk factor for atherosclerotic cardiovascular diseases, being associated with a 2.5 to 4 times higher risk of LEAD [9]. According to the guidelines for the management of arterial hypertension from the European Society of Cardiology and the European Society of Hypertension, routine use of ABI is not recommended for patients with arterial hypertension; however, it should be considered in those with moderate risk, as a positive result may reclassify them to a higher risk category [10].

People with diabetes have a two to four times higher risk of developing LEAD compared to the general population [2]. This risk increases with the severity and duration of DM, particularly when coupled with poor metabolic control and insulin use [2]. According to the guidelines on diabetes, pre-diabetes, and cardiovascular diseases from the European Society of Cardiology and the European Association for the Study of Diabetes [6], measuring the ABI can be considered a risk modifier in CVR assessment (recommendation IIb). People with DM have a higher risk of chronic ischemia, with the risk of limb involvement as the first clinical manifestation of LEAD, supporting regular screening with ABI measurement for early diagnosis. Furthermore, ABI screening is recommended at the time of DM diagnosis and after 10 years of disease, provided the initial exam results are normal (it can be considered after 5 years of diagnosis if other risk factors are present) [6].

In Portugal's primary healthcare (PHC) services, patients with arterial hypertension and/or DM have regular monitoring consultations, one of the objectives being the screening of complications such as diabetic neuropathy [11], nephropathy [12], and retinopathy [6,13]. Although these patients have a higher risk of developing LEAD, there is still no consensus on whether they should be routinely screened. Although the ABI is a simple and low-cost tool, defining the patient profile most likely to have an abnormal ABI is essential, making this screening tool more cost-effective. Family physicians are in a privileged position, not only to identify at-risk populations, but also to implement lifestyle interventions for CVR reduction.

In 2008, the Portuguese Society of Angiology and Vascular Surgery conducted a study measuring ABI in individuals over 50 in mainland Portugal, estimating a LEAD prevalence of 5.9% [14]. A subsequent study on the Portuguese island of Madeira screened individuals with DM aged 50 to 69 and those aged 40 to 49 with an additional CVR factor, and found a significantly higher LEAD prevalence of 27.1% [15]. Another study in Portugal found that 26.7% of vascular pathology-associated hospital admissions were due to LEAD, with approximately 50% occurring in emergency settings, highlighting a potential lack of timely recognition/referral by PHC services. This study also demonstrated that LEAD significantly impacts the population's morbidity and mortality and the consumption of resources in the National Health Service (NHS) [16]. 

Thus, the primary objective of this study was to assess the prevalence of asymptomatic LEAD in patients with arterial hypertension and/or DM. The secondary objective was to determine the main predictors of LEAD in this study population. As the study was conducted in at-risk groups, a higher prevalence of LEAD was expected to be detected in the studied population compared to the documented prevalence in the general population.

## Materials and methods

An exploratory, observational, and cross-sectional study was conducted on a random sample of individuals with DM and/or arterial hypertension registered in a FHU in the central region of Portugal, aged 50 to 66 years at the time of data collection. Individuals with symptoms of LEAD (such as intermittent claudication, leg pain at rest and wounds/ulcers with poor healing), those with very high CVR, amputees or those with prior revascularization, individuals with type 1 diabetes, and those unable to collaborate were excluded. Data from individual CVR stratification were calculated using the Systematic Coronary Risk Evaluation (SCORE) to estimate the 10-year risk of a fatal event, based on factors such as age, gender, smoking status, systolic blood pressure (SBP), and total cholesterol [17]. The result is expressed as a percentage and classified into risk categories: low (<1%), moderate (1-5%), high (5-10%), and very high (≥10%), and is used to guide cardiovascular prevention strategies. This assessment is integrated into SClínico, a electronic health records platform used in Portugal's health services to manage medical appointments, test results, and parametric patient data [17]. Patients with very high CVR were excluded, as the diagnosis of LEAD would not alter the therapeutic approach in the absence of symptoms. Individuals under 50 were excluded due to the lower incidence of the disease in younger populations. The upper age limit was 66, as CVR tends to be high beyond this age, making medical intervention for asymptomatic LEAD diagnosis less common.

The list of all patients with DM and arterial hypertension from the FHU was obtained through the MIM@UF database (software used by primary healthcare physicians to manage and analyze their patient lists and information from the FHU). Subsequently, those with very high CVR were manually excluded, resulting in a population of 132 with DM and 572 with arterial hypertension. Using the Raosoft sample size calculator (Raosoft Inc., Seattle, WA), with a 7% margin of error and a 90% confidence interval, the sample sizes needed to obtain a random representative sample were 112 for arterial hypertension and 68 for DM in this exploratory study. Finally, these patients were randomized using Microsoft Excel (Microsoft Corp., Redmond, WA).

ABI measurements were conducted after DM or arterial hypertension follow-up consultations. Patients were scheduled for a separate appointment if immediate measurement was not feasible. The recruitment period spanned from July 2021 to December 2022. A team of doctors, all uniformly trained, performed the measurements using standardized equipment and methods: a manual sphygmomanometer with an analog display (MDF® 840, MDF Instruments, Westlake Village, CA) and a Pocket Fetal Doppler (COMED®, Strasbourg, France) with a vascular probe. Measurements were taken with the patient in the supine position. SBP was recorded using the Doppler probe on each foot's posterior tibial artery and the arm's brachial artery. The ABI for each side was calculated by dividing the highest ankle SBP by the highest arm SBP. The estimated duration of a single ABI measurement was 15 minutes. Each side’s (left and right) ABI was assessed separately for LEAD diagnosis, with the lowest recorded ABI used for classification. ABI values were interpreted as follows: normal when ABI between 0.90 and 1.40; LEAD diagnosis when ABI ≤0.90 on at least one side; arterial incompressibility (arterial calcification) when ABI ≥1.4.

Additional variables were collected through direct patient questioning or by reviewing medical records in SClínico®. These included age, sex, body mass index (BMI), abdominal circumference, smoking history, duration of arterial hypertension and blood pressure control status, presence of dyslipidemia, most recent total cholesterol and low-density lipoprotein (LDL) levels, duration of DM, HbA1c, glomerular filtration rate (GFR) and CVR score. SClínico® has an automated tool that estimates GFR using the Cockcroft-Gault (CG) formula. However, the GFR was subsequently recalculated using the Chronic Kidney Disease Epidemiology Collaboration (CKD-EPI) equation, based on the age, sex, and serum creatinine available in the patient's medical file.

A database was created, and descriptive and inferential analyses were conducted using SPSS® software (version 26). Patients were categorized into two groups based on ABI: with and without LEAD. Categorical variables were analyzed using Fisher’s exact test. In contrast, continuous variables, which did not follow a normal distribution, were assessed using the non-parametric Mann-Whitney U test. Statistical significance was set at p<0.05.

The study received approval from the Ethics Committee of the Central Regional Health Administration of Portugal and the Coordinator and Technical Council of the FHU, where the research was conducted. ABI measurements were performed only after obtaining written informed consent from patients, ensuring participant anonymity and full compliance with data protection regulations.

## Results

Two individuals declined participation due to time constraints for the ABI measurement. The final sample consisted of 146 participants, of whom 134 had arterial hypertension and 68 had DM. Of the total sample, 56 patients had both conditions. Among the participants, 76 (52.1%) were female. The average age was 59.5±4.2 years, ranging from 50 to 66.

Regarding ABI, values ranged from a minimum of 0.6 to a maximum of 2.5, with an average of 1.1. LEAD was diagnosed in eight patients, indicating a prevalence of 5.5% within the sample. The prevalence of LEAD in participants with DM was 2.9%, while in patients with arterial hypertension was 6%. Additionally, three patients had an ABI greater than 1.4.

Regarding risk factors (see Table 1 and Table 2), 117 patients had dyslipidemia, with a median total cholesterol level of 166 mg/dL and a median LDL level of 92.9 mg/dL. Regarding BMI, 9.7% had a normal BMI, 39.6% were overweight, and 34.7% had obesity type 1. The mean abdominal circumference was 102 cm, ranging from 79 cm to 134 cm. Seven patients were identified as active smokers. Regarding CVR scores, 12.7% of patients were classified as low risk, 38.7% as moderate risk, and 48.6% as high risk. When categorized by chronic kidney disease (CKD) stage, using CG equation, the majority (n=114, 79.2%) were in stage 1, followed by 29 patients (20.1%) in stage 2 and one patient in stage 3. Among patients with arterial hypertension, 77.1% had controlled blood pressure, with an average hypertension duration of 11.7 years. In the DM group, the average duration of DM was 7.7 years, and the mean HbA1c level was 6.8%.

**Table 1:** Results of the comparison between the groups with and without LEAD (continuous variables). *Statistically significant (p<0.05). BMI: body mass index; GFR: glomerular filtration rate; CG: Cockcroft-Gault; CKD-EPI: Chronic Kidney Disease Epidemiology Collaboration; HbA1c: glycated hemoglobin; LEAD: lower extremity artery disease; LDL: low-density lipoprotein; N: absolute number. Missing values: BMI (2), abdominal circumference (23), Pack-years (16), GFR–CG equation (2), hypertension duration (18), HbA1c (1), total cholesterol (4), LDL cholesterol (4)

Parameters		LEAD	No LEAD	Total	U-value	p-value, Mann-Whitney U test
Age (years)	N	8	138	146	636.5	0.466
	Median	60	60	60		
BMI (kg/m^2^)	N	8	136	144	418	0.272
	Median	32.7	30	30.1		
Abdominal circumference (cm)	N	3	120	123	58	0.043*
	Median	115	102	102		
Pack-years (number)	N	1	129	130	7	0.123
	Median	39	0	0		
GFR (mL/min); CG equation	N	8	136	144	284.5	0.024*
	Median	140.7	109.8	111		
GFR (mL/min/1,73 m^2^); CKD-EPI equation	N	8	138	146	686.5	0.247
	Median	103	101	101.5		
Hypertension duration (years)	N	7	109	116	186.5	0.024*
	Median	20	10	10		
Diabetes mellitus duration (years)	N	2	66	68	91	0.534
	Median	4	6	6		
HbA1c (%)	N	2	65	67	51.5	0.635
	Median	6.9	6.6	6.6		
Total cholesterol (mg/dl)	N	5	137	142	184	0.079
	Median	196	166	166		
LDL cholesterol (mg/dl)	N	7	135	142	212.5	0.014*
	Median	123	90.8	92.9		

**Table 2:** Results of the comparison between the groups with and without LEAD (categorical variables). BMI: body mass index; LEAD: lower extremity artery disease; N: absolute number.; CG: Cockcroft-Gault Missing values: controlled hypertension (3), BMI (2), SCORE (4), Classes of Chronic Kidney Disease (2).

Parameters	LEAD	No LEAD	Total	p-value, Fisher’s exact test
Sex; N (%)				0.481
Female	3 (37.5%)	73 (52.9%)	76 (52.1%)	
Male	5 (62.5%)	65 (47.1%)	70 (47.9%)	
Active smoker; N (%)				0.332
Yes	1 (12.5%)	6 (4.3%)	7 (4.8%)	
No	7 (87.5%)	132(95.7%)	139 (95.2%)	
Hypertension; N (%)				1.000
Yes	8 (100%)	126 (91.3%)	134 (91.8%)	
No	0 (0%)	12 (8.7%)	12 (8.2%)	
Controlled hypertension; N (%)				0.659
Yes	5 (71.4%)	96 (77.4%)	101 (77.1%)	
No	2 (28.6%)	28 (22.6%)	30 (22.9%)	
Diabetes; N (%)				0.285
Yes	2 (25%)	66 (47.8%)	68 (46.6%)	
No	6 (75%)	72 (52.2%)	78 (53.4%)	
Dyslipidemia; N (%)				0.659
Yes	6 (75%)	111 (80.4%)	117 (80.1%)	
No	2 (25%)	27 (19.6%)	29 (19.9%)	
BMI; N (%)				0.058
Normal weight	1 (12.5%)	13 (9.6%)	14 (9.7%)	
Overweight	2 (25%)	55 (40.4%)	57 (39.6%)	
Obesity type 1	1 (12.5%)	49 (36%)	50 (34.7%)	
Obesity type 2	4 (50%)	15 (11%)	19 (13.2%)	
Obesity type 3	0 (0%)	4 (2.9%)	4 (2.8%)	
Classes of cardiovascular risk factors; N (%)				0.471
Low	1 (14.3%)	17 (12.6%)	18 (12.7%)	
Moderate	4 (57.1%)	51 (37.8%)	55 (38.7%)	
High	2 (28.6%)	67 (49.6%)	69 (48.6%)	
Classes of chronic kidney disease; N (%), CG equation				1.000
Stage 1	7 (87.5%)	107 (78.7%)	114 (79.2%)	
Stage 2	1 (12.5%)	28 (20.6%)	29 (20.1%)	
Stage 3a	0 (0%)	1 (0.7%)	1 (0.7%)	

Among the eight patients with LEAD (see Table 1-2), three were female, and five were male, with a median age of 60. Regarding comorbidities, 25% had DM, with a median HbA1c of 6.9%. All patients with LEAD had arterial hypertension, with a median duration of 20 years, and 71.4% had controlled blood pressure. Dyslipidemia was present in 75% (n=6) of the patients, with a median LDL cholesterol of 123 mg/dL and a median total cholesterol of 196 mg/dL. One active smoker was identified, with a pack-year of 39. Regarding anthropometric measures, one patient had a normal BMI, two were overweight, and five were obese, with a median abdominal circumference of 115 cm. The median GFR (using CG equation) was 140.7 mL/min, higher than that of the non-LEAD group (109.8 mL/min). When categorized by CKD stage, the majority (n=7) were in stage 1. Regarding CVR, 57.1% of patients with LEAD were classified as moderate risk.

As shown in Tables 1-2, there were statistically significant differences between the LEAD and non-LEAD groups in four variables: abdominal circumference (p=0.043), GFR using the CG equation (p=0.024), duration of hypertension (p=0.024), and LDL cholesterol (p=0.014). In all these variables, the median was higher in the LEAD group.

Some variables could not be collected for all patients due to missing or outdated information in their medical records. Notably, abdominal circumference was unavailable for 23 patients, representing a significant gap in this variable.

## Discussion

As mentioned, the primary objective of this study was to determine the prevalence of asymptomatic LEAD in a high-risk group (with DM and/or arterial hypertension). Eight patients were diagnosed with LEAD, resulting in a prevalence of 5.5%. However, comparing this result with other studies is challenging due to differences in population characteristics.

Contrary to expectations, a lower prevalence was found in this study compared to the study by Menezes (5.9%) [14]. A higher prevalence was anticipated in this study, given that it focused on high-risk groups (with DM and arterial hypertension), whereas the Menezes study assessed the general population. However, the Menezes study included individuals of advanced ages (up to 94 years), with 55.8% having hypertension and 16% having DM. It did not exclude symptomatic patients or those with very high cardiovascular risk, as in the present study.

Regarding studies on asymptomatic individuals, a multicenter study conducted in Massachusetts (United States of America) [18] screened primary care patients aged 50 to 69 years with at least one CVR factor (DM, smoking, hypertension, and/or dyslipidemia) and found an asymptomatic LEAD prevalence of 2.5%. Similarly, a study conducted in Brazil [5], involving 168 individuals aged over 55 years, with a mean age of 59.40±3.59 years (similar to that of our sample), reported a prevalence of 27.95% in asymptomatic individuals. Notably, the prevalence in the present study falls between the values reported in these two studies.

In our study, the prevalence of asymptomatic LEAD in the DM subgroup was 2.9%. A study conducted in Madeira Island on asymptomatic individuals with DM, with an age range similar to ours, reported a higher prevalence of 27.1% [15]. Significant methodological differences between the studies may explain this disparity. Unlike the Madeira study, our study excluded individuals with very high CVR and included participants with higher GFR, with only one patient (0.7%) in stage 3 chronic kidney disease (CKD). In contrast, 35.8% of patients in the Madeira study had stage 3 CKD [15]. These differences, particularly in GFR, may account for the considerable variation in prevalence rates. 

In a study conducted in Ecuador by Barrera-Guarderas et al. [19], the prevalence of LEAD in DM patients was 13.98%. This study included 578 individuals with DM, aged over 18 years (mean age 66 years), who were followed for 10 years and had at least one ABI measurement. The group diagnosed with LEAD had higher HbA1c levels, a longer duration of DM, and lower GFR. The LEAD risk was higher among women, those with a GFR <60 mL/min/m², and those using metformin combined with insulin. The average DM duration was 13.11 years. In comparison, our study sample had a shorter DM duration (7.7 years) and a higher GFR, likely due to excluding patients with very high CVR. This may explain the lower prevalence detected in our study.

The 2.9% prevalence found in the DM group in our study is notably lower than that reported in other studies. This may be attributed to the possible calcification of distal arteries, a common occurrence in DM, which can lead to falsely normal ABI values despite the presence of LEAD. Wukich et al. [20] found that 42.7% of DM patients with confirmed LEAD had normal ABI values. This suggests that ABI alone may not be sufficient for LEAD screening in DM patients. In Wukich’s study, combining ABI with the toe-brachial index (TBI) improved diagnostic accuracy, as ABI has high specificity (low false positives), while TBI has high sensitivity (low false negatives) [20].

In the hypertension group, the prevalence of asymptomatic LEAD was 6%, much lower than that reported in a study from Hungary [9]. However, Hungary is classified as a high CVR country, whereas Portugal is categorized as moderate risk. The Hungarian research by Farkas et al. [9] was a prospective multicenter observational study on asymptomatic patients with hypertension using ABI, involving 21,892 individuals aged 50-75 years (mean age 61.45 years) monitored in arterial hypertension clinics. The overall LEAD prevalence was 14.4%, with lower rates among those with controlled blood pressure (9.6% vs. 16.8%). Additionally, 8.1% of patients classified as low CVR based on SCORE and 11.1% classified as moderate CVR were reclassified based on ABI, highlighting its predictive value beyond traditional risk assessment. In that study, it was estimated that 13 patients with low CVR and 10 patients with moderate CVR needed to be assessed to identify one case of asymptomatic LEAD. The lower prevalence in our study may be due to the exclusion of very high CVR patients and the limitation of the age range to 66 years. Additionally, our sample size was significantly smaller.

Another objective of this study was to identify key predictors of LEAD in this population. The analysis revealed that abdominal obesity, assessed through abdominal circumference, was significantly more prevalent among individuals with LEAD, aligning with findings from previous studies [21]. It is worth noting, however, that only three out of the eight patients with LEAD had available data on abdominal circumference, representing a very small subgroup. This limited sample size may have influenced the results and constitutes one of the limitations of this study. As noted earlier, hypertension is a well-established risk factor for the development of LEAD [5,22]. Consequently, it is not unexpected that a longer duration of hypertension is associated with an increased risk of the disease, underscoring the importance of early detection and timely intervention. A significant association was also observed between elevated LDL cholesterol levels and the presence of LEAD, consistent with existing evidence on the role of dyslipidemia in the pathogenesis of peripheral arterial diseases [21,22]. Interestingly, a statistically significant difference in estimated GFR between the groups was observed only when using the CG formula, with the LEAD group presenting a higher median GFR. This difference was not replicated when GFR was estimated using the CKD-EPI equation. This discrepancy may be attributed to the influence of body weight on the CG formula, which is known to overestimate renal function in overweight or obese individuals [23]. In support of this, the LEAD group had a significantly higher abdominal circumference, suggesting a greater prevalence of central obesity, and a slightly higher median BMI (LEAD: 32.7 vs. No LEAD: 30). In contrast, the CKD-EPI equation, which does not incorporate body weight, produced less variability in estimated GFR values. This greater consistency may help explain the lack of statistically significant differences between the groups when this formula was applied.

It is worth noting that the low proportion of active smokers (4.8%) in our sample may not accurately represent the smoking FHU population, as smoking is a major predictor of LEAD and is frequently cited in the literature [2,4,22]. This may have influenced the calculated prevalence. Although smoking did not appear to be a determining factor for LEAD in this population, further research is needed to confirm this.

Regarding the study’s limitations, information bias may have occurred due to the use of secondary data extracted from clinical records, which may have been outdated, inaccurate, or incomplete. The involvement of multiple investigators also introduces inter-observer bias. Performing ultrasound assessments to confirm LEAD diagnosis could have helped support clinical decision-making.

Although the study sample was representative of the healthcare unit under investigation, the findings cannot be generalized to the general population. However, the prevalence found was similar to that reported by Menezes et al. [14], supporting the study’s relevance within the Portuguese context.

To our knowledge, this topic had not been previously explored in Portugal, making this study exploratory in nature. The findings provide a foundation for future research on asymptomatic LEAD in high-risk populations.

## Conclusions

In this study, the prevalence of asymptomatic LEAD in patients with DM and/or arterial hypertension from this FHU was 5.5%. Expanding the study to other health units across different regions of the country would be valuable in obtaining a larger, more diverse, and representative sample of the Portuguese population. If a significant prevalence is confirmed, implementing an organized screening program at the primary healthcare level for this population could be considered. Such a program would enable the reclassification of patients with LEAD regarding CVR, allowing for more aggressive strategies to control CVR factors. This could potentially lead to slower disease progression, as well as reduced morbidity and mortality. However, it is essential to carefully evaluate the potential health benefits against the financial investment required by the NHS to implement an organized screening. Further research is needed to assess the cost-effectiveness of screening compared to the morbidity burden associated with this disease.

This study provides the first objective data on the prevalence of LEAD in asymptomatic Portuguese patients with arterial hypertension and/or DM, offering valuable insights for healthcare planning and resource allocation for treating and preventing this condition. Raising awareness among family physicians is crucial, emphasizing the importance of early screening and diagnosis. Given their central role in primary and secondary prevention and the ease of diagnosing LEAD, family physicians are key players in improving the natural history of this disease in Portugal. The results indicate that the risk of LEAD is higher in patients with arterial hypertension and/or DM who have higher abdominal circumference, longer duration of hypertension, and higher LDL, making these individuals a priority for targeted screening. However, further studies are necessary to define the optimal screening criteria for LEAD in Portugal.
